# Estimating time-series changes in social sentiment @Twitter in U.S. metropolises during the COVID-19 pandemic

**DOI:** 10.1007/s42001-022-00186-4

**Published:** 2022-11-12

**Authors:** Ryuichi Saito, Shinichiro Haruyama

**Affiliations:** Graduate School of System Design and Management, Keio University, 4-1-1, Hiyoshi, Kohoku Ward, Yokohama City, Kanagawa Prefecture 223-0061 Japan

**Keywords:** COVID-19, Coronavirus, Twitter, Sentiment analysis, Neural network model, Transformer model, GPT-3, Location information

## Abstract

Since early 2020, the global coronavirus pandemic has strained economic activities and traditional lifestyles. For such emergencies, our paper proposes a social sentiment estimation model that changes in response to infection conditions and state government orders. By designing mediation keywords that do not directly evoke coronavirus, it is possible to observe sentiment waveforms that vary as confirmed cases increase or decrease and as behavioral restrictions are ordered or lifted over a long period. The model demonstrates guaranteed performance with transformer-based neural network models and has been validated in New York City, Los Angeles, and Chicago, given that coronavirus infections explode in overcrowded cities. The time-series of the extracted social sentiment reflected the infection conditions of each city during the 2-year period from pre-pandemic to the new normal and shows a concurrency of waveforms common to the three cities. The methods of this paper could be applied not only to analysis of the COVID-19 pandemic but also to analyses of a wide range of emergencies and they could be a policy support tool that complements traditional surveys in the future.

## Introduction

The SARS-CoV-2 coronavirus that first surfaced in Wuhan, China, in December 2019 spread globally and developed into a pandemic in 2020. As of June 26, 2022, two-and-a-half years after the outbreak, the cumulative number of cases worldwide is over 500 million, and the cumulative death toll is over 6 million. Over 5 billion people have been vaccinated with at least one dose according to the World Health Organization (WHO) [1], and vaccination is still a global primary agenda. During this time, the United States has experienced four or five waves of infection, behavioral restrictions, such as declarations of states of emergency in each state, a presidential election during the pandemic, and a national vaccination program [2]. In such emergencies, there is a need for a method that allows policymakers and public health professionals to quickly and accurately capture changes in citizens’ perceptions. For instance, if relaxing restrictions makes citizens feel more positive than policymakers expected, it may lead to the re-emergence of infections. By capturing the time-series of such perceptions, we can have a bird’s-eye view of social phenomena during the pandemic.

We propose a social sentiment estimation model for use in emergencies that is based on Twitter users located in U.S. metropolises during the pandemic. Many studies [3–7] have already attempted to estimate social sentiment in the COVID-19 pandemic, but they have the following limitations: (1) A periodic sentiment waveform that can change with the number of cases and behavioral restrictions has not been captured; (2) there have been no long-term trend analyses measuring from the pre-pandemic period to during the pandemic and then on to the new-normal period from a macro-perspective; and (3) no research has focused on large cities based on the characteristics of the coronavirus. Previous studies have evaluated text on social and news media that match keywords that directly remind us of viruses, such as “coronavirus” and “COVID-19,” so they cannot extract periodic changes in social sentiment. This is because those keywords are often used in limited contexts and emotional expressions.

In addition, according to the New York Times [8], the infection situation in the United States differs between metropolitan and rural areas, and it has been shown that since the late summer of 2020, per capita case and death rates in rural areas have outpaced those in metropolitan areas around the United States [8]. In addition, even in metropolitan areas, Rader et al. [9] have shown that the peak of the epidemic was more extreme in overcrowded cities than in less-populated cities. Therefore, when estimating social sentiment for coronavirus, it is necessary to separate metropolitan areas from rural areas and take into account cities’ sizes and characteristics. However, many previous studies have limited observational data at the linguistic, national, and state levels. To solve these problems, this research attempts the following approaches:

1. Design mediation keywords inspired by activities of citizens limited by government-issued behavioral restrictions.
2. Tweets collected based on the location information of New York City, Los Angeles, and Chicago are used as observation data from just before the pandemic to the new-normal period.
3. Estimation performance is guaranteed through transformer-based neural network techniques, such as Bidirectional Encoder Representations from Transformers (BERT) and third-generation Generative Pre-trained Transformer (GPT-3).

The time-series of the extracted social sentiment was verified by the correlation coefficient with the number of confined cases, and the feature words extracted using term frequency-inverse document frequency (TF-IDF) supported the social sentiment waveform.

One limitation to note in this study is the demographic bias of Twitter users in the United States. On Twitter, it has been found that frequent users between the ages of 18 and 49 years account for 73 % of adult users as of 2021, which diverges from the demographics of the United States [10].

The contributions of our paper are as follows:

- Proposal of a social sentiment time-series estimation model using mediation keywords that can be used during periods of emergency.
- Long-term trend analysis of U.S. metropolises, such as New York City, Los Angeles, and Chicago, and the extraction of parallel trends of social sentiment waveforms common to all three cities.
- Methodological improvements in a social sentiment estimation model using GPT-3.

The approach of this research, including keyword design, could be applied not only to the COVID-19 pandemic but also to other emergencies where citizens’ activities are restricted. In addition, by deploying and operating the model of this research on a data-streaming platform, it is possible to capture the time-series data of social sentiment in real-time emergencies.

## Literature review

### Coronavirus and natural language processing

Since January 2020 and the global spread of the coronavirus, many attempts have been made to use natural language processing methods to extract social insights from text information exchanged through the internet. First, Kruspe et al. [3] studied social sentiment during the pandemic using the neural network method. Kruspe et al. extracted social sentiment from Twitter in European countries, such as Italy, France, and Spain, during the initial months of the pandemic using a Multilingual Universal Sentence Encoder [11]. Caliskan [12] selected Ohio as a state with less ideological bias in the United States and multilaterally estimated tweets’ emotions using the GloVe [13] and Bidirectional Recurrent Neural Network (RNN) models. Chakraborty et al. [12] primarily indexed sentiment for a news-article dataset of the Global Database of Events, Language, and Tone (GDELT) project [14] using the AFINN Sentiment Lexicon and examined the relationship between the number of cases and deaths in China, the United States, Italy, and India. Saleh et al. [6] estimated the sentiment of tweets matching #socialdistancing and #stayathome sent between March 27 and April 10, 2020, using the AFINN Sentiment Lexicon, and then attempted to cluster topics through Latent Dirichlet Allocation (LDA). Abd-Alrazaq et al. [5] classified English tweets that match keywords, such as “corona” and “COVID-19”, into 12 topics primarily using LDA and scored sentiment by topic using the Python library TextBlob. Ridhwan et al. [7] evaluated sentiment on Twitter during the pandemic period of February through August 2020 in Singapore using both neural network-based RNN [15] and lexicon-based Valence Aware Dictionary and sEntiment Reasoner (VADER) [16] approaches. Moreover, Hussain et al. [17] visualized changes in citizens’ susceptibility to vaccines on Facebook and Twitter from March through November 2020 in the United Kingdom and the United States using VADER and BERT [18].

Our research takes a different approach from the above-mentioned methods. Previous studies have inferred the sentiment of texts from social networking services and news media that match keywords relevant to coronavirus and behavioral restrictions. However, these keywords are often only used in a limited context, so it can be difficult to capture periodic waves in tandem with increases or decreases in case numbers and the addition or relaxing of behavioral restrictions. In this study, we try to solve the above limitations by focusing on the sentiment of citizens limited by the behavioral restrictions issued by the state government.

### Transformer-based neural network model

Next, we give an overview of the transformer-based neural network model on which this study relies. Transformer [19] is a model that solves the difficulty of parallelizing the training of RNN models [15, 20, 21] based on a two-part network of encoders and decoders [22] to handle tasks with different input and output lengths, such as machine translation and chatbots. The transformer uses an attention mechanism instead of a lengthy recursive network. The attention mechanism is inspired by the human eye, and can learn the relationship between distant tokens and between sentences by investigating the similarities between word vectors.

The BERT [18] language model uses Transformer’s encoder and has demonstrated state-of-the-art performance in language-understanding evaluation. BERT trains in two phases: pre-training and fine-tuning. In the pre-training phase, the attention mechanism trains a huge dataset to construct a general-purpose model, and in the fine-tuning phase, the model is adjusted according to the actual application. The pre-training phase uses two steps, Masked Language Modeling (MLN) and Next Sentence Prediction (NSP), to train sentences bidirectionally using the attention mechanism. In the fine-tuning phase, the parameters obtained by the pre-training phase are used as the initial values of the weights, and the training is specialized for the question.

GPT-3 [23] is a language model that uses Transformer’s decoder, and it was developed to support general-purpose tasks with only pre-training operation by 175 billion parameters. The model architecture of GPT-3 inherits GPT-2 [24], which was based on GPT [25]. GPT-3 achieves higher accuracy than GPT-2 by training with a larger dataset extracted from Common Crawl and Web-Text2. It has been confirmed that GPT-3 accomplishes high accuracy without fine-tuning, but in this study, we tried fine-tuning to realize even higher accuracy. In this research, we support the reliability of the estimation results using the above transformer-based neural network methods.

## Methods

Initially, to extract the transition of social sentiment from the pre-pandemic period to the new-normal period, tweets inspired by citizens’ activities limited by restrictions in New York City, Los Angeles, and Chicago were retrieved from December 30, 2019, to January 2, 2022. The retrieved tweets were classified into sentiment using a neural network model that was fine-tuned on the Twitter dataset and indexed numerically. The indexed sentiment was validated for correlation with the number of confirmed cases, and then, feature words were identified using the TF-IDF to confirm the trend of tweets classified into sentiments.

### Data collection

Tweets were collected using the Twitter application programming interface (API) and aggregated by type of behavioral restriction.

#### City and timeframe

Coronavirus infections in the United States have grown at different speeds in metropolitan and rural areas depending on the time of year [8], and it has been confirmed that infections tend to explode in overcrowded cities rather than in less-populated cities [9]. New York City, Los Angeles, and Chicago were selected as observation targets for this research based on their respective populations and the number of tweets sent in those cities. In the U.S., New York City, Los Angeles, and Chicago are the most congested cities in terms of population according to U.S. Census Data [26] and in terms of the number of tweets for each city according to Förster et al. [27].

In the actual search, the Full-archive Search API of Twitter API v2 was used to collect tweets posted within a 25-mile radius of each city’s city hall. The 25-mile radius setting was based on the Full-archive Search API limit, but we consider this reasonable for collecting tweets from the center of these large cities.

Our search period was the 2-year period from December 30, 2019, to January 2, 2022, capturing sentiment from before the coronavirus pandemic to the new normal following repeated outbreaks and behavioral restrictions. In addition, tweets were aggregated weekly to offset the weekend effect.

#### Keywords

Previous studies [3–7] used keywords that directly relate to coronavirus or behavioral restrictions to estimate citizens’ sentiment during the pandemic; however, these methods have the following two limitations.

1. It is not possible to compare the pre-pandemic period with the pandemic period, because these keywords were either not recognized by the public before the pandemic or were used in other ways.
2. These keywords are often used in a negative context and cannot be compared to the infection-spread period and convergence period in tandem with infection status.

**Fig. 1:**
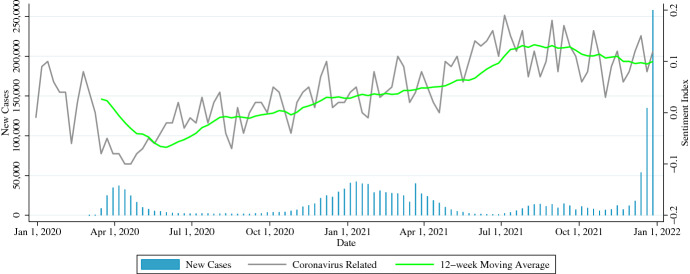
Sentiment Index of Tweets that Match Keywords Related to Coronavirus in New York City

**Fig. 2:**
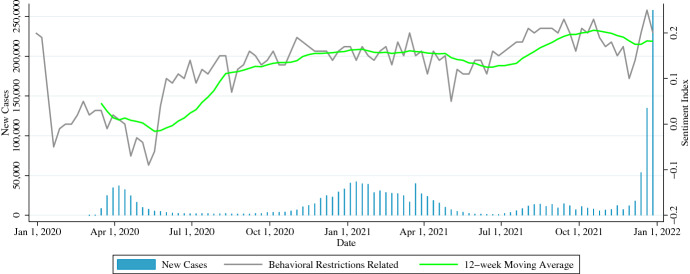
Sentiment Index of Tweets that Match Keywords Related to Behavioral Restrictions in New York City

Figures 1 and 2 show the results of estimating the time-series of sentiment in New York City using keywords relevant to the coronavirus and behavioral restrictions. The keywords used are shown in Table 1. These keywords were designed based on previous studies, Centers for Disease Control and Prevention (CDC) usages [28], and similarities between words using Word2Vec [29, 30]. In addition, the BERT model described in Section (1) later was used for sentiment estimation.^1^ The higher the value of the sentiment index, the more negatively it is interpreted and vice versa.

In Fig. 1, the sentiment index value ranges between −0.05 to 0.1 during the pre-pandemic period when the coronavirus was not well recognized by the public, but it drops to less than −0.05 during the first outbreak in April 2020. In addition, the sentiment index ranges between 0.05 and 0.2 after April 2021, which is generally considered to be the time when behavioral restrictions were lifted and citizens looked ahead to the new normal. Figure 2 confirms the same trend as Fig. 1. From the above, these keywords are not appropriate for capturing social sentiment, such as fear and anxiety about the spread of infection or a sense of security about the end of infection.

**Table 1:** Keywords Related to Coronavirus and Behavioral Restrictions

Coronavirus-related keyword	Behavioral restriction-related keyword
	emergency, stay-at-home
coronavirus, virus, covid	stayathome, social distancing
covid-19, sars-cov-2, pandemic, epidemic	socialdistancing, lockdown, shutdown
	curfews, quarantine, mask

The word “corona” was often used in the context of drinking during the pre-pandemic period and, therefore, was excluded from the coronavirus-related keywords

We focus on the activities of citizens limited by behavioral restrictions. These include common activities, such as commuting, dating, and traveling, even during the pre-pandemic period. By designing keywords based on such activities, we hypothesized that we could extract social sentiment according to the number of cases and the status of behavioral restrictions. These keywords are related to coronavirus and behavioral restrictions, but are also used in contexts outside of coronavirus or behavioral restrictions. In other words, they are the keywords that mediate between coronavirus and inference. In addition, we proposed mediation keywords inspired by the activities of citizens limited by government restrictions.

In Hallas et al. [31], as part of the response of the U.S. state government to the pandemic, the transition of restrictions was investigated by dividing into three types: containment and closure, economic response, and health systems. The present study designed mediation keywords based on citizens’ activities as limited by this containment and closure, which represents behavioral restrictions. In Hallas et al., containment and closure were subcategorized into eight restrictions, such as workplace closings and restrictions on gathering size, but in the present study, we recategorized them into three restrictions, as shown in Table 2. Keywords that are restricted activities were designed according to the CDC’s descriptions [28] for Stay-at-home-ordered and Travel Restrictions and to detailed Alabama State orders [32], as well as the CDC description for Restrictions on Gatherings. Also, since the meaning of a single word changes depending on the context, we designed phrases consisting of two or three words related to state-level restrictions.

**Table 2:** State-level restrictions and mediation keywords

No.	State-level regulations		Mediation keywords	
1	Stay-at-Home-Ordered		go(-ing) out	to college
			go(-ing) to work	attend the class
			to my office	by train
			to school	by bus
2	Restrictions-on-Gatherings	Public Event	to the movies	gathering
			to the theater	to a club
			see a play	to the amusement$^{1}$
			to a (the) concert	go bowling
			to the gig	to casinos
			to the museum	to the racetrack
			to the planetarium	to the bingo$^{2}$
			to the auditorium	to a (the) arcade
			see the game	to church
			to the stadium	
		Private Event	wedding	to a bar
			on a date	get-together
			to a (the) party	drinking party
			have a party	to a restaurant
			to bbq	eat(-ing) out
			have a bbq	to a café
			for a drink	meetup
		Non-Essential	go(-ing) shopping	go swimming
		Retail	to the mall	to a (the) spa
			to the salon	to yoga
			get a haircut	to massage
			to the gym	
3	Travel-Restrictions		travel(-ing)	by plane
			on a trip	visit
			fly to	to my hometown

$^{1}$ Assuming “to the amusement parks”

$^{2}$ Assuming “to the bingo halls”

To estimate the sentiment of citizens limited by behavioral restrictions, the collected tweets did not need to have been posted in the context of coronavirus or behavioral restrictions. This is because our study does not estimate opinions on coronavirus and behavioral restrictions as in the results of Figs. 1 and 2, but rather estimates the daily emotional changes that citizens have in both normal and pandemic periods. On the other hand, changes in the overall theme exchanged in the weekly aggregated tweet space are supported by the TF-IDF as described in a later section. Finally, retweet information was excluded from the search results.

#### Collection result

The tweets obtained by the Twitter API will be explained. The total number of tweets is 309,425, the number of unique users is 102,807,^2^, and the total file size is 88.4 M bytes. Table 3 shows the number of tweets, and Table 4 shows the number of unique users in each city for each restriction type. Also, Table 5 indicates the number of tweets for each number of words in the keyword. As originally expected, using phrases with fewer words located a higher number of tweets.

**Table 3:** Number of tweets by restriction type

	New York City		Los Angeles		Chicago	
Restriction type	Tweets	Pct	Tweets	Pct	Tweets	Pct
Stay-at-Home-Ordered	42,724	29.9 %	39,878	33.0 %	14,733	32.0 %
Restrictions-on-Gatherings	34,941	24.5 %	27,840	23.1 %	7,507	16.3 %
Travel-Restrictions	64,999	45.6 %	52,950	43.9 %	23,853	51.7 %
Total	142,664	100.0 %	120,668	100.0 %	46,093	100.0 %

**Table 4:** Number of unique users by restriction type

	New York City		Los Angeles		Chicago	
Restriction types	U.U	Pct	U.U	Pct	U.U	Pct
Stay-at-Home-Ordered	19,013	30.8 %	19,545	33.9 %	7,120	31.9 %
Restrictions-on-Gatherings	16,180	26.2 %	14,829	25.7 %	4,306	19.3 %
Travel-Restrictions	26,514	43.0 %	23,362	40.5 %	23,853	48.8 %
Total	61,707	100.0 %	57,736	100.0 %	22,331	100.0 %

U.U stands for Unique Users

**Table 5:** Number of tweets by keyword word count

	New York City		Los Angeles		Chicago	
Keyword word count	Tweets	Pct	Tweets	Pct	Tweets	Pct
One Word	65,025	45.6 %	52,310	43.4 %	24,770	53.7 %
Two Words Phrase	46,501	32.6 %	41,161	34.1 %	14,120	30.6 %
Three Words Phrase	31,138	21.8 %	27,197	22.5 %	7,203	15.6 %
Total	142,664	100.0 %	120,668	100.0 %	46,093	100.0 %

In this study, tweets were retrieved from June 17 to August 30, 2022. According to Yoshida [33], when Yoshida attempted to retrieve tweets based on tweet IDs in the public Twitter dataset associated with COVID-19, they reported that 15.3% of all tweets were inaccessible. Accordingly, in our study, it should be noted that we may not have retrieved all tweets from December 29, 2019, to January 2, 2022.

### Training inference

In this study, a transformer-based neural network model is tested as a sentiment-inference method. Although there is a method to classify sentiment based on a pre-registered dictionary, this method cannot classify texts that do not match the dictionary. For the results filtered in advance by keywords, such as in Table 2, classification based on contextual information using a neural network is appropriate.

#### Neural network model and fine-tuning

In this study, we used the BERT and the GPT-3 models based on Transformer architecture. In the BERT model, BERT-Base [34], which was pre-trained on a dataset consisting of English Wikipedia and 11,038 unpublished books, was used. As a machine learning library, Pytorch 1.7.1 and torchtext 0.8.1 were used for training and inference processes. The number of epochs in training and validation tasks was 14. In GPT-3, Open AI GPT-3 Curie [35], which is a model optimized for language translation, complex classification, text sentiment, and summarization, was used.

For training data, the Sentiment 140 dataset [36], which labeled tweets with emotions, was used for fine-tuning. After removing the URL and mention information starting with @ from the Sentiment140 tweet dataset, the positive and negative data were divided equally, and then, the dataset was divided into 80% training data and 20% validation data. The split data are shown in Table 6.

**Table 6:** Fine-tuning data excerpted from sentiment140

	BERT			GPT-3		
	Positive	Negative	Total	Positive	Negative	Total
Training data	178,706	178,706	357,412	17,870	17,870	35,740
Validation data	44,677	44,677	89,354	4,467	4,467	89,34

#### Accuracy of models

In addition to the Sentiment140 dataset used for training, the datasets collected were used for testing, and then, the collected datasets were manually labeled. For labeling, an author and two collaborators labeled sentiment for the same data and a majority vote determined the final label for the test data. The test results are shown in Table 7. Although the GPT-3 training data are approximately 1/10 of the BERT training data, the accuracy of the BERT model was 77.1% and the GPT-3 model was 89.5% in the Sentiment140 dataset. Furthermore, in the collection dataset, the accuracy of the BERT model was 72.4% and the GPT-3 model was 81.0%. This result confirms the superiority of GPT-3’s performance in the sentiment classification of Twitter tweets.

**Table 7:** Accuracy of models using test data

	BERT		GPT-3	
	Number	Accuracy	Number	Accuracy
Sentiment 140 dataset	4,964	77.1 %	4,964	89.5 %
Collection dataset	1,014	72.4 %	1,014	81.0 %

### Methods of indexing

The retrieved tweets were converted to sentiment using a classifier application that implemented the BERT and GPT-3 models. In the BERT classifier, the accuracy of the model could only be guaranteed at 72.4% in the collection dataset, so tweets with a probability estimation result of less than 0.70 were sent to neutral sentiment. Each tweet in the BERT was classified as 0 for positive, 1 for neutral, or 2 for negative, and 0 was normalized to –1, 1 to 0, and 2 to 1 to make the neutral 0. In the GPT-3 classifier, the accuracy of the model was sufficiently guaranteed to be 81.0% in the collection dataset, so positive was defined as 0 and negative was defined as 2 for polar classification, and finally, 0 was normalized to –1 and 2 was normalized to 1. Tables 8 and 9 show examples of sentiment classified and indexed by a classifier application that implemented the BERT and GPT-3 models. The results are the arithmetic mean of the classified values of the sentiment index, aggregated on a weekly basis from Monday to Sunday. The higher the value of the index, the more pessimistic the sentiment is throughout the week, and the lower the value, the more optimistic the sentiment.

**Table 8:** Sample of classified tweets by BERT

Tweets	Index	Sentiment
yes i went to the gym twice before coronavirus came to my town. So	– 1	Positive
I am ready to fight		
Yes. It’s doable but less ideal. And the nyc coffeeops meetup just	0	Neutral
switched to online-only for this week		
I have a feeling my coworkers are gonna cancel the plans we have and	1	Negative
I’m gonna be upset because I wanna go out		

Sampled from classification results for the week of March 9, 2020 in New York City

**Table 9:** Sample of classified tweets by GPT-3

Tweets	Index	Sentiment
my fiancé’s and my wedding is next Saturday and our honeymoon was	– 1	Positive
at Disney but we’ve always enjoyed going to snow whites wishing well		
I hope the coronavirus is gone by the summer I’m trying to visit the	1	Negative
motherland, I miss my grandma!		

Sampled from classification results for the week of March 9, 2020 in Los Angeles

### Corroboration of index

To confirm the usefulness of the extracted sentiment index, it is validated in two ways. First, a correlation coefficient^3^ is used to examine the time-series relationship between the extracted sentiment and the number of cases. A significantly higher value of the correlation coefficient indicates a higher sensitivity of citizens to the number of infected cases in each city during that period, and vice versa.

The second is the extraction of feature words of classified tweets using TF-IDF. TF-IDF is a feature vectorization method widely used to identify the importance of terms to document in the corpus^4^, and feature words are used to identify unique words of tweets extracted on a weekly basis. If many feature words related to coronaviruses are extracted, it is interpreted as many tweets being exchanged in the context of coronaviruses, and vice versa.

The extracted words characterize the entire theme exchanged during that period. In this research, we have already filtered tweets using detailed keywords in Table 2, so feature words are extracted using the TF-IDF method, not topic extraction such as LDA.

## Results

This section shows a time-series of the sentiment of citizens limited by the restrictions of each metropolis of New York City, Los Angeles, and Chicago from December 30, 2019, to January 2, 2022. This section focuses on a time-series analysis of the sentiment index within these same regions. The indexes extracted using GPT-3, which showed high accuracy, were drawn, and the indexes extracted using BERT were drawn as a reference. For the number of new infections, the New York Times COVID-19 Data [37] categorized at the county level as of April 3, 2022 were used and aggregated weekly. The correlation coefficient was used to confirm the relationship between sentiment index and infection status, and the 4-week average analysis was completed in addition to the weekly analysis. Furthermore, each city’s timeline and feature words were referenced to ascertain the relevance of the sentiment index to events, such as state government orders.

### New York City

**Fig. 3:**
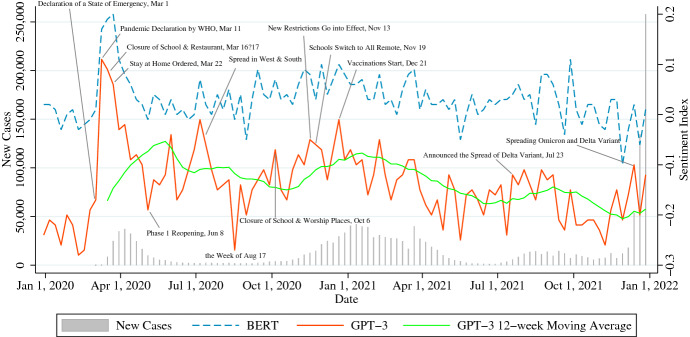
Sentiment Index in New York City

New Cases on the left axis of Fig. 3 show the number of cases identified in New York City, and the right axis shows the index of sentiment extracted by the sentiment classifier. By January 2022, New York City had experienced four waves of infection in the spring and winter of 2020 and the fall and winter of 2021. In New York State, unlike in the South and West, there was no spike in infections in the summer of 2020, and since July 2020, California has surpassed New York in terms of the number of infected cases.^5^

**Table 10:** Correlation coefficient between GPT-3 Sentiment Index and new cases in New York City

	Feb 24, 2020–July 6, 2020			Sep 7, 2020–May 31, 2021		
Lag	–	1 week	2 weeks	–	1 week	2 weeks
Total	0.26	**0**.**63**	**0**.**83**	0.31	**0**.**49**	**0**.**56**
*p value*	0.26	0.00	0.00	0.05	0.00	0.00
Stay-at-Home-Ordered	0.11	0.43	**0**.**72**	0.31	**0**.**46**	**0**.**41**
*p value*	0.65	0.06	0.00	0.06	0.00	0.01
Restrictions-on-Gatherings	$-$0.06	0.18	0.28	**0**.**45**	**0**.**49**	**0**.**53**
*p value*	0.80	0.44	0.44	0.00	0.00	0.00
Travel-Restrictions	0.14	**0**.**48**	**0**.**74**	0.20	**0**.**36**	**0**.**44**
*p value*	0.55	0.03	0.00	0.23	0.03	0.01
Total 4-week Average	**0**.**55**	**0**.**81**	**0**.**91**	**0**.**46**	**0**.**61**	**0**.**72**
*p value*	0.01	0.00	0.00	0.00	0.00	0.00

	Jul 28, 2021–Oct 25, 2021			Nov 1, 2021–Dec 27, 2021		
Lag	–	1 week	2 weeks	–	1 week	2 weeks
Total	0.07	0.36	0.40	0.45	0.31	**0**.**79**
*p value*	0.79	0.14	0.10	0.22	0.42	0.01
Stay-at-Home-Ordered	**– 0.51**	$-$0.21	$-$0.18	0.55	0.56	0.59
*p value*	0.03	0.39	0.48	0.13	0.11	0.09
Restrictions-on-Gatherings	$-$0.09	0.30	0.10	0.65	**0**.**73**	0.53
*p value*	0.73	0.22	0.70	0.06	0.02	0.14
Travel-Restrictions	0.18	0.11	0.35	0.36	0.25	**0**.**67**
*p value*	0.47	0.66	0.16	0.35	0.52	0.05
Total 4-week Average	0.22	0.40	**0**.**48**	**0**.**75**	**0**.**76**	**0**.**92**
*p value*	0.37	0.10	0.04	0.02	0.02	0.00

Lag refers to the time lag in the time-series of the number of the sentiment index; if it is listed as 1 week, the number of cases against the sentiment index is delayed by 1 week

Total 4-week average represents the correlation coefficient between the 4-week average of the number of cases and the 4-week average of the sentiment index

Bold values indicate significance at the 5% significance level in a two-sided test

First, we describe the plotted waveform of sentiment. The correlation coefficient between the BERT sentiment and GPT-3 sentiment is 0.77.^6^ Table 10 shows the correlation coefficient between the sentiment index obtained and the number of confirmed cases. Lag means the time lag in the number of cases against the sentiment index. For instance, 2 weeks refer to the correlation coefficient with the number of cases 2 weeks after the week in which the sentiment index was extracted. In Table 10, Total means the total sentiment extracted for each restriction type, and a positive correlation is confirmed in the lag of 1 week to 2 weeks until May 2021, but no significant correlation is confirmed after July 2021. Additionally, from examining the correlation coefficient by restriction type, we can see that tweet sentiment by type is associated with the number of confirmed cases in the lag of 1 week to 2 weeks until May 2021. On the other hand, the total 4-week average as a trend line confirms a significant positive correlation throughout the period from February 2020 to December 2021, and in each period, the total 4-week average values show a higher correlation than the weekly values.

**Table 11:** Feature words for negative sentiment in New York City

Weeks	Feature Words
Dec 30, 2019	nye, war, eve, gray, lana, trump, support, ww0, brady, barry
Jan 6, 2020	understate, latinx, misleading, equal, students, chosen, graduate, maintain, systems
Mar 9, 2020	**ban**, **coronavirus**, europe, **virus**, trump, hanks, uk, schools, **cancelled**
Mar 16, 2020	**virus**, **coronavirus**, **quarantine**, **essential**, **closed**, trump, **distancing**, **corona**
Jun 29, 2020	**mask**, rushmore, **covid**, july, **virus**, bothdir, trump, vía, stunningly
Jul 6, 2020	**covid**, trump, devos, betsy, goya, threatens, schools, **reopening**, **masks**, risk
Aug 17, 2020	**covid**, **pandemic**, station, summonses, beyfulhu, mamma, violating, street
Nov 23, 2020	thanksgiving, **covid**, nate, holiday, christmas, **pandemic**, holidays, health
Nov 30, 2020	**covid**, elena, nuff, robin, christmas, dec, **pandemic**, gee, activists, esu, naira
Dec 14, 2020	snow, av, christmas, winter, storm, **covid**, chito, **mask**, birx, cold
Dec 21, 2020	christmas, uk, **strain**, iggy, **covid**, santa, holidays, cynthia, trump
Feb 1, 2021	snow, shovel, snowstorm, blizzard, **covid**, storm, restricted, plow
Apr 19, 2021	kedar, pulmonologist, faridabad, nit, **icu**, delhi, **hospital**, ncr, litres
Apr 26, 2021	tcoew, **pandemic**, tsakos, mwah, **mask**, scholar, temples, sore, customer
Aug 2, 2021	**vaccinated**, dipset, **vaccine**, **covid**, **vaccines**, **variant**, proof, busway, **delta**
Aug 16, 2021	**vaccinated**, **covid**, hurricane, afghanistan, storm, fen, **mandate**, diving
Dec 20, 2021	christmas, **covid**, **omicron**, lucas, holiday, **tested**, marley, **test**, **symptoms**
Dec 27, 2021	nye, citymd, **covid**, **omicron**, **positive**, **pcr**, marsh, windham, **tests**, eve

Words in bold are words relevant to the coronavirus. The feature words of weeks with high sentiment value and weeks with low sentiment value as mentioned in this study are emphasized

**Table 12:** Feature words for positive sentiment in New York City

Weeks	Feature words
Dec 30, 2019	decade, nye, eve, january, lexus, incall, central, en, hustles, resolution
Jan 6, 2020	january, bacch, todayi, winter, livingston, resolution, rug, waitress, booth
Mar 9, 2020	**coronavirus**, melted, **ban**, **corona**, **virus**, defending, trump, hanks, uk
Mar 16, 2020	saracen, **distancing**, **coronavirus**, temporary, hyatt, **virus**, lauren
Jun 29, 2020	july, fireworks, **mask**, quiz, **covid**, rave, **pandemic**, brook, hair
Jul 6, 2020	trump, zucchini, hardcore, vía, july, **mask**, nytimes, hostels, **covid**
Aug 17, 2020	polls, mma, submission, formplease, sony, entry, submit, monthly, alpha
Nov 23, 2020	thanksgiving, **covid**, holiday, turkey, **mask**, hasidic, zelda, fitzgerald
Nov 30, 2020	**covid**, colish, kearnian, dec, christmas, holiday, thanksgiving, december
Dec 14, 2020	snow, santa, siedc, holiday, holidaymembership, kemper, eloise, claus, winter
Dec 21, 2020	christmas, merry, holidays, holiday, eve, santa, dansdeal, pell, stockings
Feb 1, 2021	snow, bijous, feb, blizzard, storm, corinthians, devotionals, snowy
Apr 19, 2021	fling, whale, exam, yuh, apathy, **vaccinated**, spring, earth, sue, props, **vaccine**
Apr 26, 2021	savage0, **vaccinated**, mars, futuristic, pops, brook, heed, church0, pregnant
Aug 2, 2021	august, gt, regina, doge, kinsey, rajeswaran, liamwhen, fryers, jha
Aug 16, 2021	cashtag, rting, facey, eastwood, hewlett, homework, henri, completed, manor
Dec 20, 2021	christmas, merry, holiday, **omicron**, eve, **covid**, playmates, holiday, xmas
Dec 27, 2021	**covid**, eve, nye, playmates, christmas, spliff, **test**, olaf, paintingplease

Next, we verify the sentiment waveform. In Fig. 3, sentiment spiked in the week of March 9, 2020, and continued to peak until the week of March 23. The first cases of infection were confirmed in New York State on March 3, after which Governor Cuomo announced the New Rochelle containment area on March 10 and WHO declared a global COVID-19 pandemic on March 11.^7^ As of March 25, the number of infected cases in New York accounted for more than 7% of the total number of cases worldwide, and Governor Cuomo stated that the closure of schools and gatherings dramatically delayed the exponential increase in infections.^8^ In this first wave of infections from March, the peak of sentiment index values overlaps with the above period. Table 11 and 12 shows the top feature words extracted by the TF-IDF regarding sentiment. These feature words support that, during this first wave, negative tweets using keywords related to Stay-at-home-ordered Restrictions, Restrictions on Gatherings, and Travel Restrictions were exchanged in contexts related to COVID-19 using words such as “coronavirus,” “canceled”, “quarantine”, “essential,” and “distancing”.

In the summer of 2020, infections subsided in New York City, and the sentiment index rose to 0.00 by the week of July 6, 2020. In the feature words of the same period, shown in Table 11, the keywords related to behavioral restrictions decreased, while those related to coronavirus were still prevalent. Infection spikes in the South and West, rather than in New York City, might have affected the city’s sentiment index. In the second wave, citizens’ awareness may have risen, as Governor Cuomo tightened regulations on schools and places of worship on October 6 in response to increasing cases in parts of New York City.^9^ Then, through November, behavioral restrictions increased as hospitalization rates broke records.^10^

According to Fig. 3, the value of the sentiment index decreased from the week of November 23 to the week of November 30, but according to the feature words in Tables 11 and 12, there is a high possibility that the Thanksgiving holidays had an effect. In addition to the spread of coronavirus infection, as seen in the feature words, the winter storm that occurred in mid-December might have contributed to the rise in the sentiment index in December.^11^ (A similar winter snowstorm effect was confirmed from feature words in the week of February 1, 2021.) It should be noted that holidays make citizens feel positive about going out, gatherings, and travel, and storms are natural behavioral restrictions; therefore, our study’s keywords were sensitive to these events. In addition, focusing on the feature words of positive sentiment in late April 12, when the number of cases and the sentiment index decreased, the topic of vaccination increased.

After June 2021, no significant correlation between the number of cases and the sentiment index could be confirmed, except for the 2-week time lag in the winter wave of 2021. However, as can be seen from the feature words in Table 11, it is highly possible that tweets related to coronavirus affected the sentiment index even during this period. In particular, keywords related to vaccination from the week of August 2 to 16, 2021 and keywords related to Omicron from the week of December 20 to 27 stand out.

### Los Angeles

**Fig. 4:**
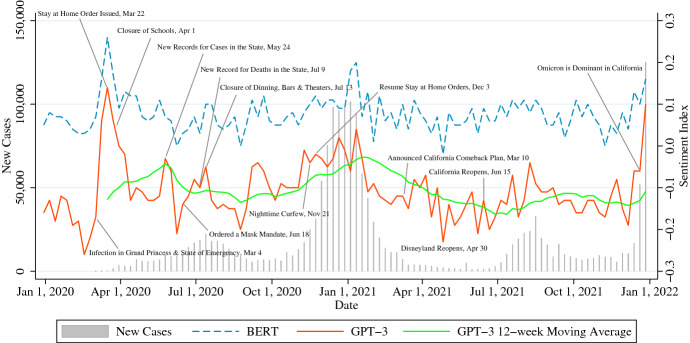
Sentiment in Los Angeles

The left axis of Fig. 4 shows the number of infection cases in Los Angeles County, and the right axis shows the sentiment index. Unlike New York State, California has experienced five waves of infection, including in the summer of 2020, with Los Angeles County having the highest cumulative number of confirmed cases at the county level in the United States in June 2020 [38]. Additionally, in California, since Governor Newson issued a stay-at-home order in March 2020, he has been intermittently adding and easing behavioral restrictions, eventually announcing re-opening on June 15, 2021, without capacity restrictions or distancing requirements.^12^

**Table 13:** Correlation coefficient between Sentiment Index and New Cases in Los Angeles

	Feb 24, 2020–Apr 6, 2020			Apr 13, 2020–Aug 31, 2020		
Lag	–	1 week	2 weeks	–	1 week	2 weeks
Total	0.22	0.64	**0**.**95**	0.23	$-$0.01	$-$0.18
*p value*	0.63	0.12	0.00	0.32	0.98	0.44
Stay-at-Home-Ordered	0.31	0.63	**0**.**82**	0.34	0.27	0.08
*p value*	0.51	0.13	0.02	0.13	0.24	0.74
Restrictions-on-Gatherings	0.08	0.59	**0**.**85**	$-$0.18	$-$0.05	$-$0.15
*p value*	0.86	0.16	0.02	0.44	0.83	0.52
Travel-Restrictions	$-$0.02	0.44	**0**.**80**	$-$0.11	$-$0.02	$-$0.16
*p value*	0.97	0.33	0.03	0.63	0.92	0.48
Total 4-week Average	**0**.**78**	**0**.**94**	**0**.**97**	$-$0.35	**– 0.45**	**– 0.55**
*p value*	0.04	0.00	0.00	0.12	0.04	0.01

	Oct 12, 2020–Mar 15, 2021			Jun 14, 2021–Oct 11, 2021		
Lag	–	1 week	2 weeks	–	1 week	2 weeks
Total	**0**.**73**	**0**.**73**	**0**.**74**	**0**.**59**	**0**.**68**	0.34
*p value*	0.00	0.00	0.00	0.01	0.00	0.17
Stay-at-Home-Ordered	0.17	0.19	0.21	$-$0.45	$-$0.17	$-$0.24
*p value*	0.43	0.38	0.32	0.06	0.49	0.33
Restrictions-on-Gatherings	**0**.**47**	0.30	0.22	0.19	0.34	0.29
*p value*	0.03	0.16	0.31	0.45	0.17	0.24
Travel-Restrictions	**0**.**76**	**0**.**75**	**0**.**71**	**0**.**55**	**0**.**63**	0.54
*p value*	0.00	0.00	0.00	0.01	0.01	0.11
Total 4-week Average	**0**.**84**	**0**.**88**	**0**.**85**	**0**.**93**	**0**.**85**	**0**.**67**
*p value*	0.00	0.00	0.00	0.00	0.00	0.00

**Table 14:** Correlation coefficient between Sentiment Index and New Cases in Los Angeles

	Nov 15, 2021–Dec 27, 2021		
Lag	0 week	1 week	2 weeks
Total	**0**.**89**	0.63	0.48
*p value*	0.01	0.13	0.27
Stay-at-Home-Ordered	0.20	**0**.**91**	0.25
*p value*	0.67	0.00	0.59
Restrictions-on-Gatherings	0.45	**0**.**92**	0.64
*p value*	0.32	0.00	0.12
Travel-Restrictions	**0**.**87**	0.48	0.61
*p value*	0.00	0.16	0.24
Total 4-week Average	**0**.**97**	**0**.**79**	**0**.**78**
*p value*	0.00	0.04	0.04

Lag refers to the time lag in the time-series of the number of the sentiment index; if it is listed as 1 week, the number of cases against the sentiment index is delayed by 1 week

Total 4-weeks Average represents the correlation coefficient between the 4-week average of the number of cases and the 4-week average of the sentiment index

Bold values indicate significance at the 5% significance level in a two-sided test

First, we explain the sentiment waveform. The correlation coefficient between the BERT sentiment and GPT-3 sentiment is 0.71.^13^ Also, from Tables 13 and 14, we can see the significant values as compared to other cities, not including the second wave. In particular, the keywords associated with Travel Restrictions are positively correlated with cases except the second wave. Furthermore, even after June 2021, when socioeconomic activities resumed, the correlation between the number of cases and the sentiment index continued to be confirmed, which is a unique feature of Los Angeles.

Then, the sentiment waveform is verified. The first wave began with an infection on the cruise ship Grand Princess on the week of March 2, 2020, and in the same week, Governor Newsom declared a State of Emergency [39]. The number of cases peaked 2 weeks later during the week of March 30. According to Table 13, the correlation coefficient is 0.95 in total with a lag of 2 weeks, and the correlation coefficient for each restriction type is also a significant value. Keywords such as “cancel”, “quarantine”, “lockdown”, and “closed” also stand out from Table 15, which confirms that negative tweets posted in the context of behavioral restrictions contributed to the waveform.

**Table 15:** Feature words for negative sentiment in Los Angeles

Weeks	Feature words
Dec 30, 2019	nye, fakes, brady, decade, trump, sick, overcome, january, war, monkeys, holiday
Jan 6, 2020	lt, australia, game, gt, iran, dumpsters, 0am, testify, iraq, bed
Mar 9, 2020	**coronavirus**, **virus**, europe, **ban**, **corona**, trump, **cancelled**, sick, **cancel**
Mar 16, 2020	**virus**, **quarantine**, **lockdown**, **corona**, **coronavirus**, **closed**, **quarantined**
Jul 6, 2020	**covid**, **pandemic**, **mask**, trump, schools, **virus**, children
Jul 13, 2020	**mask**, **covid**, children, **masks**, send, lewis, oc, **distancing**, **pandemic**, sending
Aug 24, 2020	**covid**, trump, **pandemic**, debt, **ban**, smiley, fahk, convoluted, campaign
Aug 31, 2020	**covid**, trump, pelosi, heat, stylist, salon, cemetery, salons, reed, allowed, **pandemic**
Dec 21, 2020	**covid**, christmas, holiday, **pandemic**, são, fret, argentines, pearls, paulo
Dec 28, 2020	**covid**, nye, **pandemic**, **quarantine**, icus, holiday, news, partying, **hospitals**
Jun 14 2021	swimming, carlon, bookshop, play, overtime, desmond, expedia, summer, supposed
Jun 21 2021	uo, vp, kamala, border, harris, remaining, vino, scheduler, alienating, campanile
Aug 9, 2021	**vaccinated**, **covid**, twellman, children, asked, apy, 0psi, unprompted, hamm
Aug 16, 2021	onlyfans, fcn, garner, takeover, **covid**, **vaccinated**, webapp, momster, begrudgingly
Dec 20, 2021	christmas, **covid**, **omicron**, **sick**, holidays, rise, nuclear, betty, rain
Dec 27, 2021	nye, betty, omarion, **covid**, rain, eve, **boosted**, molly, resolution, **omicron**, vaxxed

Bold letters are words that are reminiscent of coronavirus

The feature words of the week with high sentiment value and the week with low sentiment value mentioned in the paper are mainly selected

**Table 16:** Feature words for positive sentiment in Los Angeles

Weeks	Feature words
Dec 30, 2019	penn, decade, inventory, playdays, thursdays, collectibles, whittier, street
Jan 6, 2020	ponder, diaz, flawlessweddingsandevents, blowout, repped, josh, inquiries
Mar 9, 2020	penn, inventory, whittier, thursdays, **coronavirus**, playdays, collectibles
Mar 16, 2020	penn, inventory, thursdays, **quarantine**, whittier, **distancing**, playdays
Jul 6, 2020	safety, gloves, playdays, collectibles, **precautions**, **mask**, using, hotwheels
Jul 13, 2020	safety, **mask**, collectibles, playdays, gloves, **covid**, **precautions**, using
Aug 24, 2020	faculty, safety, daej, hydro, sacred, virtual, **mask**, waistbeads
Aug 31, 2020	labor, yogis, 0pcollaborative, safety, wed, playdays, september
Dec 21, 2020	christmas, holidays, holiday, merry, xmas, dermot, mum, eve, bruin, cardini
Dec 28, 2020	noa, agentphillipsor, illuminati, phillips, 0email, whatsapp, bang, alec
Jun 14 2021	unofficial, ath, june, elite, wr, star, malachi, associate, dei
Jun 21 2021	unofficial, star, antwi, kojo, exceeded, sategna, usc, receiver, summer
Aug 9, 2021	proctor, tribe, jc, chickens, august, buenos, vallarta, selling, meet
Aug 16, 2021	denim, yada, ranger, gt, august, staff, ek0, lorri, barley, janssen, **unvaccinated**
Dec 20, 2021	christmas, holiday, holidays, fare, january, departing, gift, liberia, segment
Dec 27, 2021	nye, sole, **vaccinated**, **boosted**, laverne, bora, fits, commit, preston

In the next wave of infections from spring to summer of 2020, infection numbers peaked during the week of July 13. In the following week, the number of cases in California exceeded New York State, reaching the highest level in the United States.^14^ In Los Angeles, no significant correlation was found between confirmed cases and the sentiment index from spring to summer, 2020. According to Table 15, tweets on the theme related to “mask” were prevalent. By June, Governor Newsom had announced that Californians would be required to wear face masks in public,^15^ and public awareness had likely risen, coupled with increased infections (Table 16).

In the spread from October 2020, infections peaked from December 2020 to January 2021. In addition, the total correlation coefficient has a high value of 0.73 to 0.74, and unlike in the other two cities, there is a possibility that citizens’ awareness was highly sensitive regarding coronavirus during this period in Los Angeles. The high level of public interest in the coronavirus can be seen from the feature words of the same period in Table 15. According to Tables 13 and 14, the correlation for both the June 14 to October 11 and November 15 to December 27 periods is significant, although there are differences in the time lag. Citizens may have been more concerned about the coronavirus during both the pandemic and new-normal periods in Los Angeles than in the other two cities, according to their feature words and correlation coefficients.

### Chicago

**Fig. 5:**
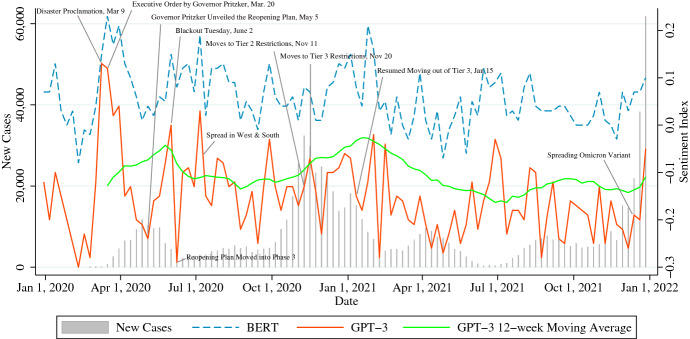
Sentiment in Chicago

New Cases in Fig. 5 display a weekly time-series of the number of cases in Cook County, Illinois, where the city of Chicago is located. Citizens of Chicago have experienced four major waves of infection from 2020 to 2021 at the same times as New York City.^16^ Interestingly, Illinois consistently had the lowest levels of cases and deaths in the country from late spring to early summer of 2020. However, the city also faced the highest level of deaths per week of all states during the second surge in the winter of 2020.^17^

First, we can describe the sentiment waveform. The correlation coefficient between the BERT sentiment and GPT-3 sentiment is 0.76.^18^ On the other hand, a strong correlation between the sentiment index and the number of cases could not be confirmed, as shown in Table 17. During the Omicron wave, which started in October 2021, the correlation coefficient of 0.87 in total shows a strong positive correlation only when the lag between the sentiment index and cases is not set.

**Table 17:** Correlation coefficient between Sentiment Index and new cases in Chicago

	Feb 24, 2020–Jun 8, 2020			Sep 28, 2020–Feb 25, 2021		
Lag	–	1 week	2 weeks	–	1 week	2 weeks
Total	$-$0.42	$-$0.29	0.38	0.04	$-$0.22	0.10
*p value*	0.10	0.28	0.14	0.87	0.35	0.69
Stay-at-Home-Ordered	$-$0.41	$-$0.35	$-$0.14	$-$0.11	$-$0.18	$-$0.04
*p value*	0.12	0.18	0.60	0.66	0.45	0.85
Restrictions-on-Gatherings	0.18	0.24	$-$0.12	0.22	0.06	0.14
*p value*	0.50	0.37	0.65	0.34	0.81	0.56
Travel-Restrictions	**– 0.54**	$-$0.44	0.04	0.26	$-$0.06	0.09
*p value*	0.03	0.09	0.89	0.26	0.81	0.71
Total 4-week Average	$-$0.30	$-$0.04	0.18	0.08	0.07	0.18
*p value*	0.26	0.89	0.50	0.74	0.77	0.44

	Jul 5, 2021–Sep 27, 2021			Oct 25, 2021–Dec 27, 2021		
Lag	–	1 week	2 weeks	–	1 week	2 weeks
Total	$-$0.15	$-$0.35	$-$0.33	**0**.**87**	0.61	0.34
*p value*	0.63	0.23	0.27	0.00	0.06	0.34
Stay-at-Home-Ordered	$-$0.34	**– 0.58**	**– 0.68**	0.20	0.27	0.34
*p value*	0.25	0.04	0.01	0.57	0.46	0.34
Restrictions-on-Gatherings	0.19	0.11	$-$0.22	**0**.**71**	0.61	0.06
*p value*	0.54	0.73	0.47	0.02	0.06	0.86
Travel-Restrictions	$-$0.09	$-$0.29	$-$0.20	**0**.**72**	0.22	**– 0.87**
*p value*	0.76	0.34	0.52	0.02	0.55	0.00
Total 4-week Average	**– 0.66**	**– 0.66**	**– 0.58**	**0**.**87**	0.31	$-$0.46
*p value*	0.01	0.01	0.04	0.00	0.38	0.19

Lag refers to the time lag in the time-series of the number of cases against the time-series of the sentiment index; if it is listed as 1 week, the number of cases against the sentiment index is delayed by 1 week

Total 4-week average represents the correlation coefficient between the 4-week average of the number of cases and the 4-week average of the sentiment index

Bold values indicate numbers determined to be significant at the 5% significance level in a two-sided test

Second, the sentiment waveform is verified. The sentiment waveform’s peak in March 2020, shown in Fig. 5, could be explained by the announcement of behavioral restrictions by Governor Pritzker. Orders issued on March 20 required citizens to stay at home and non-essential businesses to be closed statewide, which restriction was extended until May 29, 2020.^19^ On May 5, 2020, Governor Pritzker announced a re-opening plan consisting of Phases 1–5,^20^ and in the same week, sentiment responded positively. Additionally, the negative reaction in sentiment in early June may have been heavily influenced by posts about Blackout Tuesday, according to Table 18.

**Table 18:** Feature words for negative sentiment in Chicago

Weeks	Feature words
Dec 30, 2019	nye, tonight, tomorrow, refill, promotion0, iran, scream, cleaned
Jan 6, 2020	literally, bigfoot, kindred, peñuelas, files, tired, east, index, jared
Mar 9, 2020	**coronavirus**, **ban**, **virus**, **quarantine**, st, cheap, patrick, booked, **closings**
Mar 16, 2020	vote, **quarantine**, groceries, **coronavirus**, tomorrow, primary, colombia
Jun 1, 2020	protest, protesters, police, black, looters, protesting, deli, ninis, white
Jun 8, 2020	anhorrhism, calumet, reduced, mcdonalds, differently, beach, chuck, race
Nov 9, 2020	**gatherings**, thanksgiving, sleazy, preachin, **mask**, **lockdown**, **covid**
Nov 16, 2020	thanksgiving, **covid**, selfish, **virus**, forgo, staying, debt, partying
Nov 23, 2020	thanksgiving, holiday, **covid**, dumped, flown, suggest, rise, rebeccaszajna
Jan 11, 2021	cpd, portraits, aht, feds, insulting, irony, health, say, security, propaganda
Jan 18, 2021	shaming, coat, venom, perimeter, meters, claim, lightning, inauguration, indoor
Jun 7, 2021	hot, coo, devastation, cleaning, zoe, shady, wanda, stranger, staring
Jun 14, 2021	kd, juneteenth, corpus, marginalized, quite, choice, blah, wyoming, rod
Aug 9, 2021	cursing, radioactive, pelvic, cavity, finances, vaginal, ministry, cancer
Aug 16, 2021	frosting, afghanistan, **vaccinated**, psu, drawls, havnt, advisory, tmw
Dec 20, 2021	**covid**, christmas, **positive**, bacon, **vaccinated**, **tested**, evac, plummer
Dec 27, 2021	nye, betty, **covid**, snow, **boosted**, **tests**, rapid, **test**, **cdc**, **negative**, **vaccinated**

Bold letters are words that are reminiscent of coronavirus

The feature words of the week with high sentiment value and the week with low sentiment value mentioned in the paper are mainly selected

**Table 19:** Feature words for positive sentiment in Chicago

Weeks	Feature words
Dec 30, 2019	folo, stands, accounts, rt, election, tweet, decade, shall, win, lauren
Jan 6, 2020	insta_chicago, chicago_city_insta, reba, safehouse, southport, lakeith
Mar 9, 2020	spring, paper, **virus**, **corona**, govt, sanitizer, break, **hand**, cheap, maga, **wash**
Mar 16, 2020	vote, local, stay, worksheets, online, **quarantine**, gt, **virus**, wfh
Jun 1, 2020	protest, malmborg, black, protesters, websitevisit, purchased, democratic
Jun 8, 2020	agirlaboutchicago, _gothamcity, democratic, hair, tequila, abstracts, roberson
Nov 9, 2020	cellist, liberty, thanksgiving, november, internet, services, spoken, free
Nov 16, 2020	paleta, thanksgiving, holiday, gpu, bfb, lance, arami, benson, ssd, nonsmoking
Nov 23, 2020	thanksgiving, empowers, trendsetter, holidays, doing, tree, cudahy, dinkins
Jan 11, 2021	dobby, fbi, pines, january, winter, chapel, onigiri, giza, kororin, pockets
Jan 18, 2021	softball, **tier**, **mitigations**, aloud, framing, ced, ciara, **ban**
Jun 7, 2021	booking, policy, summer, compete, business, text, limo, cce, taleju, number, cats
Jun 14, 2021	brow, mahomes, imnotart, brady, sew, aiw, kenneth, hairs, father, jagoff, endorsed
Aug 9, 2021	berwyn, crim, event, sunglasses, std, rugby, rector, walk, weather, lu
Aug 16, 2021	dawes, empire, grants, nourishing, ua0, cps, event, prevention, partners, gately
Dec 20, 2021	christmas, xmas, merry, santa, holiday, wb, grinded, orthopaedic
Dec 27, 2021	nye, schwarma, dmo, tipped, bible, winter, taco, losing, **testing**, wage, greg

During the second wave, which began in November 2020, the restriction level moved into Tier 2 on November 11 and then into the highest Tier 3 on November 20 in response to a surge in cases.^21^ Sentiment responded negatively as the restriction level shifted. Then, toward the end of November, sentiment responded positively, and feature words in November 2020 of Table 19 show that this was the effect of the Thanksgiving holidays. In addition, on January 15, 2021, mitigations at Tier 3 restriction levels were resumed,^22^ and in Table 19, the keywords “mitigations” and “tier” stand out as positive words.

For 2020, we could not confirm the correlation between the sentiment index and the number of cases in Chicago, but we could indicate an association between the orders and relaxing of behavioral restrictions by the State government, although the relationship between behavioral restrictions and sentiment time-series was disrupted by Blackout Tuesday and the Thanksgiving holidays. On the other hand, for 2021, the relationship between the sentiment index and coronavirus-related events was not confirmed, which could have been due to the lifting of orders and restrictions.

## Discussion

While the Results section analyzed the relationship between the number of infections and sentiment trends within each region, this section attempts to compare sentiment waveforms in each city. First, as a general trend of the waveform common to all three cities, we could see that sentiment spiked in the first wave of infection and then gradually stagnated over time. In each figure, the sentiment index exceeded 0.1 in March 2020, and after that, we found that sentiment gradually declined according to 12-week moving average lines. Hallas et al. [31] demonstrate the time-series of the index, showing that stringency of policy response was relaxed toward December 2020, despite increasing infections per capita in each state of the United States. Although there are other possible factors, such as vaccinations and reduced lethality of mutant strains, it is conceivable that relaxing mitigation policies might have turned around the sentiment of citizens limited by behavioral restrictions. Chakraborty et al. [12] show that the degree of negativity reduced over time in English news articles related to the coronavirus within a short period of 60 days; therefore, the influence of change in news media could be considered at the same time. Furthermore, compared to the pre-pandemic period, the sentiment index in January 2020 was around $-$0.2 in each city, but it was still higher in New York City and Los Angeles after April 2021 when citizens became conscious of the new normal. In these cities, sentiment affected by behavioral restrictions might not have returned to pre-pandemic levels even in the latter half of 2021.

Table 20 shows the correlation coefficients between the sentiment indexes of each city. Significance is shown at the 5% significance level in the two-sided test between cities. In particular, a strong relationship between the New York City and Los Angeles was confirmed, and a parallel trend of waveforms was observed between each city. In the stringency index by Hallas et al. [31], New York and California have been considered relatively strict States in their policy response to the pandemic, but in terms of infection status, Los Angeles is different from New York City, for example, in experiencing a wave of infections in the summer of 2020. On the other hand, as seen in Tables 10, 13, and 14, the significance of the correlation between the number of cases and the sentiment index varies with the observation period in New York City and Los Angeles. From the above, we can see that the sentiment waveform of each city was not solely influenced by its own infection situation.

**Table 20:** Correlation coefficient between sentiment indexes in each city

	Los Angeles	Chicago
New York City	0.71	0.60
*p value*	1.40E-17	0.00
Chicago	0.62	–
*p value*	1.70E-12	–

## Limitations

There are three limitations to this study. The first is the limitation of the sentiment classification model. The BERT model was classified into three polarities (positive, neutral, and negative) based on the probability of inference, but in the GPT-3 model, it could not return inference probabilities due to the specifications of API. More accurate results could be derived by creating Twitter training data classified into three polarities.

The second limitation regarded the extracted sample. In the study, tweets posted were evaluated, but the number of tweets retrieved has decreased over time^23^ A decrease in the number of tweets might indicate a decrease in citizens’ interest, and the characteristics of the sample population might have changed over time. Therefore, in the future, a multifaceted approach such as an evaluation of user bias should be used.

The third limitation regards the sentiment captured. In this study, we proposed a social sentiment estimation model set in three U.S. metropolises during the COVID-19 pandemic period from December 30, 2019, to January 2, 2022. However, in New York City, the influences of the winter storm in December 2020 and the blizzard in February 2021 were also confirmed from the extracted feature words. Events that limit citizen behavior, such as natural disasters, also bring pessimistic feelings to citizens, just like the coronavirus. However, the results of this experiment also suggest that the social sentiment estimation model of this study could be applied to natural disasters.

## Conclusion

We proposed a social sentiment estimation model based on Twitter use in New York City, Los Angeles, and Chicago during the coronavirus pandemic. By designing mediation keywords that are related to the coronavirus, but do not explicitly mention the coronavirus, we could estimate sentiment in response to infection numbers and level of behavioral restrictions. In addition, these estimation results are verified by the performance of the transformer-based GPT-3 model. And, using these results, we were able to capture long-term trends in the sentiment of citizens in large cities during a pandemic for the first time in history. In our results, the correlation between the sentiment index and infection numbers differed for each city. In Los Angeles, a relatively positive correlation between the sentiment index and the number of cases was confirmed over 2 years, but the same was not confirmed in Chicago. On the other hand, in each city, the relationship between the timeline of events related to COVID-19 and the waveform was confirmed, and this result was supported by feature words using TF-IDF. In addition, we identified concurrency between the New York City and Los Angeles waveforms, suggesting a general and universal trend in citizens’ sentiment during this period.

Our model is applicable not only in COVID-19 pandemic situations but also in general emergencies that restrict the activities of citizens, such as natural disasters. Furthermore, estimating the time-series of social sentiment in an emergency from a macro-perspective will allow us to confirm the periodicity and inertia of the sentiment wave at that time. In addition, implementing these estimation models on data-streaming platforms has the potential impact to be applied to policymakers’ understanding of citizen sentiment in policy-making and feedback after policy implementation in an emergency.

## Figures and Tables

**Fig. 6:**
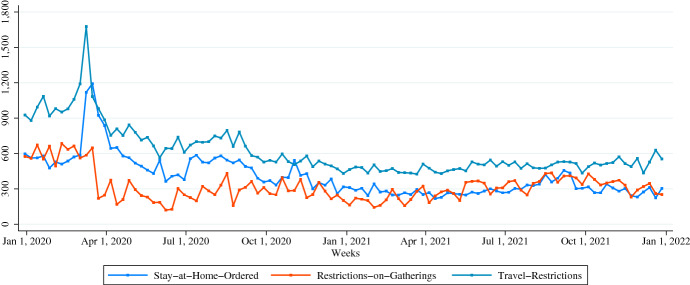
Retrieved tweets in New York City

**Fig. 7:**
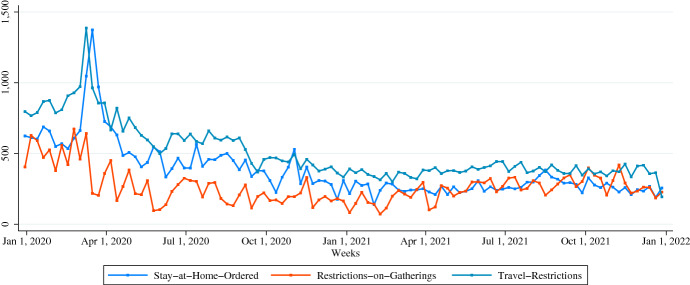
Retrieved tweets in Los Angeles

**Fig. 8:**
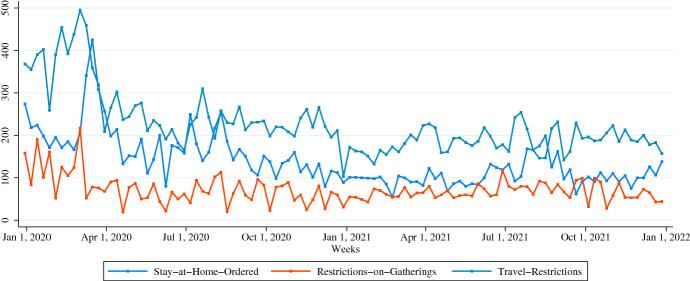
Retrieved tweets in Chicago

## Data Availability

The data generated during the current study are available in the GitHub repository, https://github.com/RyuichiSaito1/covid19-twitter-usa.

## Appendix

Figures 6, 7, and 8 show the time series of the number of tweets retrieved for each type of state-level restriction. In all three cities, tweets related to Stay-at-home-ordered and Travel Restrictions peaked during the weeks of March 2 to March 16, 2020, and tended to decline thereafter. Regarding tweets related to Stay-at-home-ordered and Travel Restrictions, there were fewer collections after June 2020 than between January and February 2020. Also, by restriction type, the number of tweets related to Travel Restrictions was the highest throughout this period, followed by Stay-at-home-ordered Restrictions and Restrictions on Gatherings.

Table 21 shows the summary statistics of weekly 105 values generated by indexing tweets retrieved from December 30, 2019, to January 2, 2022, in each city.

**Table 21:** Summary statistics of sentiment index

City	Model	Obs	Mean	Std. Dev	Min	Max
New York City	BERT	105	0.03	0.05	$-$0.10	0.20
	GPT-3	105	$-$0.14	0.07	$-$0.28	0.11
Los Angeles	BERT	105	0.08	0.04	$-$0.02	0.26
	GPT-3	105	$-$0.11	0.07	$-$0.26	0.14
Chicago	BERT	105	0.05	0.06	$-$0.09	0.23
	GPT-3	105	$-$0.15	0.09	$-$0.30	0.12

## Footnotes

1 BERT was used, because GPT-3, described in a later section, has API usage and cost limitations.

2 This value does not match the total in Table 4, because this value is the result of deduplication between restriction types.

3 The covariance represents the strength of the linear relationship between two data, but the correlation coefficient is a numerical measure to be standardized to take the range of –1 to 1 with the standard deviation of each data, as the covariance depends on the size of the value of the data.

4 TF-IDF is denoted by the product of Term Frequency *TF(t,d)* and Inverse Document Frequency *IDF(t,D)*. *t* is the term, *d* is the document, and *D* is the corpus.

5 Los Angeles Times: California has the most COVID-19 cases in the U.S., surpassing New York, as the spike continues. Accessed on 03.25.2022 (2020). https://www.latimes.com/california/story/2020-07-22/california-has-most-covid-19-infections-of-any-state-as-surge-continues.

6 p value: 5.68E-22.

7 The New York Times: ‘Containment Area’ Is Ordered for New Rochelle Coronavirus Cluster. Accessed on 06.20.2022 (2020). https://www.nytimes.com/2020/03/10/nyregion/coronavirus-new-rochelle-containment-area.html.

8 The New York Times: De Blasio Calls Stimulus Deal’s Treatment of N.Y.C.‘Immoral’. Accessed on 03.25.2022 (2020). https://www.nytimes.com/2020/03/25/nyregion/coronavirus-new-york-update.html.

9 The Wall Street Journal: New York Can Stop the Second Wave of Covid-19, Gov. Cuomo Says. Accessed on 03.25.2022 (2020). https://www.wsj.com/articles/new-york-can-stop-a-second-wave-of-covid-19-gov-cuomo-says-11602534486.

10 The New York Times: As U.S. Breaks Hospitalization Records, N.Y. and the Other States Add Restrictions. Accessed on 06.25.2022 (2020). https://www.nytimes.com/live/2020/11/11/world/covid-19-coronavirus-live-updates.

11 The New York Times: This Is Not the Way New Yorkers Normally Greet a Major Snowstorm. Accessed on 06.25.2022 (2020). https://www.nytimes.com/2020/12/17/nyregion/nyc-winter-storm-pandemic.html.

12 The New York Times: A Timeline of the Coronavirus in California. Accessed on 06.25.2022 (2020). https://www.nytimes.com/2021/06/15/us/coronavirus-california-timeline.html.

13 p value: 3.10E-17.

14 Los Angeles Times: California has the most COVID-19 cases in the U.S., surpassing New York, as the spike continues. Accessed on 03.25.2022(2020). https://www.latimes.com/california/story/2020-07-22/california-has-most-covid-19-infections-of-any-state-as-surge-continues.

15 Los Angeles Times: Californians must wear face masks in public under coronavirus order issued by Newsom. Accessed on 03.25.2022(2020). https://www.latimes.com/california/story/2020-06-18/california-mandatory-face-masks-statewide-order-coronavirus-gavin-newsom.

16 The New York Times: Tracking Coronavirus in Illinois: Latest Map and Case Count. Accessed on 06.25.2022(2022). https://www.nytimes.com/interactive/2021/us/illinois-covid-cases.html.

17 the Chicago Tribune: Gov. Pritzker, don’t go soft in the fight against COVID-19. Accessed on 06.18.2022 (2022). https://www.chicagotribune.com/opinion/commentary/ct-opinion-illinois-coronavirus-pritzker-greising-20201210-m63t3l7dcvbcdn44gmx7alnvt4-story.html.

18 p value: 0.00.

19 State of Illinois: Executive Order 2020–10. Accessed on 06.18.2022(2020). https://www.illinois.gov/government/executive-orders/executive-order.executive-order-number-10.2020.html.

20 State of Illinois: RestoreIllinois Accessed on 06.18.2022(2020). https://coronavirus.illinois.gov/restore-illinois.html.

21 State of Illinois: Actions to Combat a Resurgence of COVID-19. Accessed on 06.18.2022(2020). https://coronavirus.illinois.gov/restore-illinois/mitigation-plan.html.

22 State of Illinois: Restore Illinois Introduction. Accessed on 06.18.2022(2020). https://coronavirus.illinois.gov/restore-illinois/introduction.html.

23 Refer to Figs 6, 7, and 8 in Appendix.
